# Exploration of optical fibres as a carrier for new benzene and toluene matrix-free reference materials

**DOI:** 10.1007/s00216-015-8758-3

**Published:** 2015-05-15

**Authors:** Marta Słomińska, Mariusz Marć, Jolanta Szczygelska-Tao, Piotr Konieczka, Jacek Namieśnik

**Affiliations:** Faculty of Chemistry, Department of Analytical Chemistry, Gdańsk University of Technology, G. Narutowicza 11/12, 80-233 Gdańsk, Poland; Faculty of Chemistry, Department of Chemistry and Technology of Functional Materials, Gdańsk University of Technology, G. Narutowicza 11/12, 80-233 Gdańsk, Poland

**Keywords:** Reference materials, Quality assurance, Quality control, Organic compounds, Trace organic compounds

## Abstract

To meet high expectations concerning precision and accuracy of reference materials, preparation of matrix-free reference materials using thermal decomposition-gas chromatography-mass spectrometry (TD-GC-MS) was proposed in this study. In the paper, the results obtained in preparation of the new reference materials for benzene and toluene are presented, based on the thermal decomposition technique of compounds chemically bound to the surface of optical fibre segments. The results obtained at various stages of the research procedure (homogeneity, stability) confirmed the possibility of using prepared laboratory samples of materials as reference materials for benzene and toluene. For the prepared batch of materials, reference values 1.26 ± 0.91 (ng/fibre) for benzene and 11.3 ± 7.4 (ng/fibre) for toluene were determined.

## Introduction

Reference materials play a role of property value carriers determined for the applied specific material. Thus, by evaluating the suitability of a reference material, the following criteria should be taken into consideration [1–5]:

- *Homogeneity of the material*. The reference material should be sufficiently uniform in the various parts of the material so that the value(s) refers to the properties of any other batch of reference material.
- *Stability of the material*. A reference material and the value(s) characterizing its properties should be stable at a certain time and under storage, transport and usage conditions.
- *Precision and accuracy of determining the value of a reference material property*. The property value should be determined using a method burdened with a slight systematic error and using measuring instruments or measures of material that can be linked to national standards of measurement units and thus also consistent with similar units in other countries.
- *Documentation of reference material*. There should be a clear description of the material’s characteristics, the methods of determining the values of the reference material, and the value of the measurement’s uncertainty of the property value. Certification and possession of a certificate prepared in accordance with ISO Guide 31 recommendations are an additional advantage of a reference material.

Given the above guidelines, it can be concluded that the ability to collect and transmit information about the material properties results from the requirements of reference materials.

Many scientific-technical institutions whose activities relate to metrological issues are involved in the production and certification of various kinds of reference materials. The most important institutions involved in the manufacture of gaseous reference materials include the following:

- *IRMM i IAEA* (Europe)
- *National Institute of Standards and Technology* (*NIST*) (USA)
- *NRCC* (Canada)
- *IChTJ* (Poland)

Also, in other institutions, attempts are made to manufacture various types of reference materials. They are often focused on a specific type of material. The largest producers of certified gaseous mixtures are *Linde Industrial Gases* and *Messer Group* (Germany). The choice of a suitable reference material depends on the type of analytical task, the characteristics of the test material, the applied analytical procedure and the expected analytical information obtained in the course of the measurements.

Due to the fact that one-ring aromatic hydrocarbons play a significant role in the chemistry of the atmosphere and have a harmful effect on living organisms, they are a common element of laboratory research conducted worldwide [6–11]. Benzene and its alkylated derivatives (toluene, ethyl benzene, xylenes) belong to the group of volatile organic compounds (VOCs), which are structurally the simplest among aromatic hydrocarbons but which have a significant impact on the environment. This is due to the fact that environmental pollution, which includes benzene, toluene and xylene (BTX) group compounds, plays a key role in the formation of secondary contamination of the environment and thus is involved in [12, 13]

- The formation of photochemical smog (ozone + oxidants)
- Intensification of the greenhouse effect

The danger associated with benzene and its alkylated derivatives in the environment is subject to their toxic, mutagenic and carcinogenic effects on living organisms. Additionally, benzene, toluene, ethyl benzene and xylene (BTEX) group compounds constitute a risk not only to the environment but also to the public health. Therefore, they are the most important pollutants considered by environment protection organizations.

Intensive development of analytical techniques for measurement of gaseous media and the negative effects (mutagenic, carcinogenic and neurotoxic) of BTX and oxygenated organic compounds make it particularly important to monitoring and determination of these compounds in the air. The problem is the availability of gaseous reference materials.

Nowadays, the reference materials dedicated for the analysis of BTX compounds are generally available in two forms:

- As a standard gaseous mixtures in cylinders
- As a form of sorptive tubes with the following sorbing agents [14, 15]:
  - Tenax (*CRM 112*), the reference material for BTEX compounds
  - Active charcoal, the reference material for benzene toluene and m-xylene

Many research institutions lead the projects dedicated for preparation of new types of reference materials. NIST and Virginia Tech (VT) have conducted the research on the prototype of reference material that emits a single volatile organic compound such as toluene, analogously to the emissions of a diffusion-controlled building product [16].

On the other hand, the Brazilian Metrology Institute (Inmetro) has developed a certified reference material for BTEX compounds in methanol [17].

The worldwide research projects are focused on searching for new solutions which enable to prepare homogenous, stable and handy reference materials for volatile organic compounds.

The principle of preparation of standard gas mixture using thermal decomposition (TD) process is based on controlled decomposition of surface compound in strictly defined temperature during the specified period of time. TD process results in the formation of the analyte stream which is mixed with the stream of diluting gas forming the stream of standard gas mixture [18].

Thermal decomposition of surface compounds is characterized by many advantages due to the preparation of VOC reference materials. The possibility of application in case of toxic, volatile and reactive analytes and the production of single component and multi components of standard mixtures with a broad and controlled range of concentrations give possibility to apply a TD-GC-MS technique for preparation of reference materials for benzene and toluene.

## Experimental

**Fig. 1:**
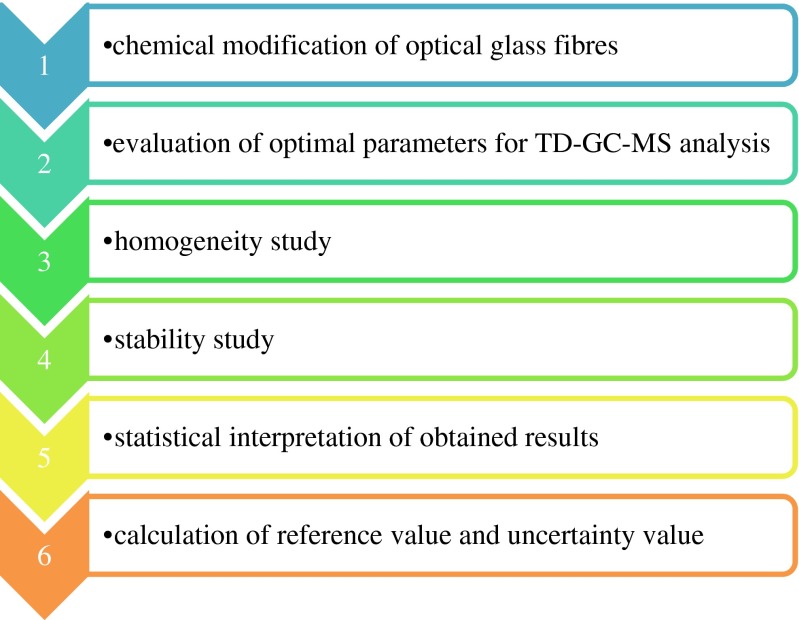
Research procedure for the preparation of new matrix-free reference materials for benzene and toluene

The research procedure is shown in Fig. 1.

### Chemical modification of glass fibres

The process of chemical modification of glass fibres has been developed and carried out by a team from the Department of Chemical Technology, Faculty of Chemistry, University of Gdańsk. The starting material for the process of glass fibre chemical modification was coated with a thin layer of aluminium (inner fibre diameter/outer fibre diameter ratio 660:680 μm).

**Fig. 2:**
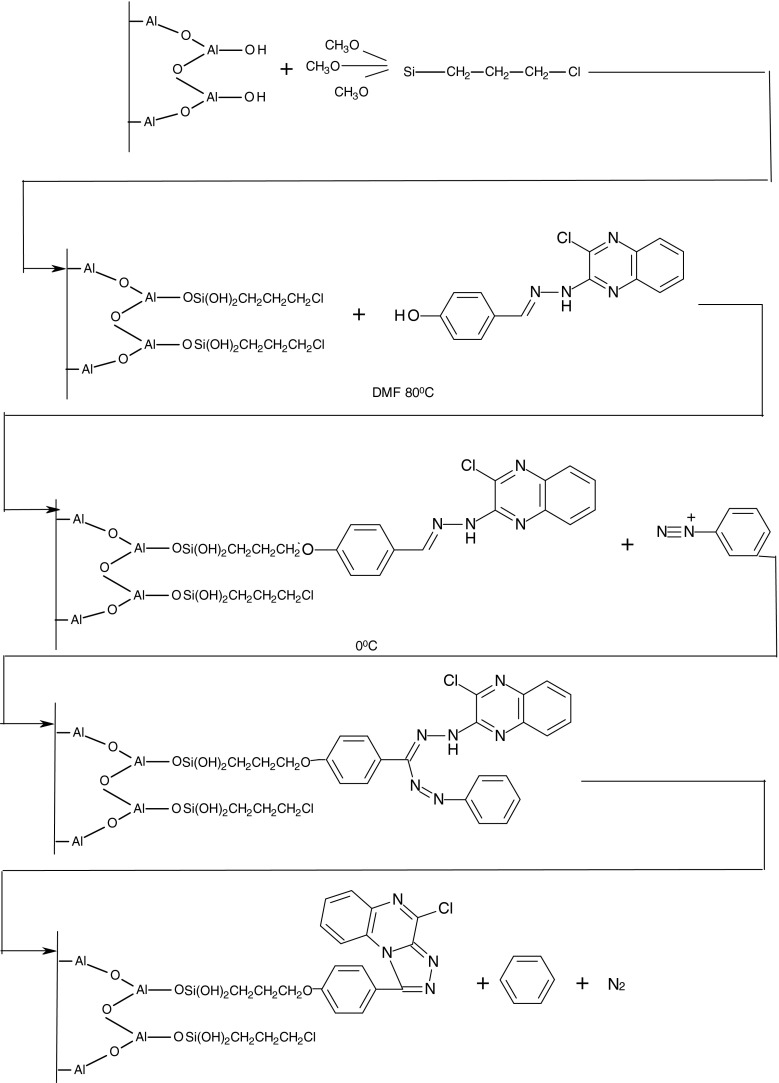
Diagram of the surface glass fibre chemical modification process in order to form a surface compound acting as a source of benzene in the thermal decomposition process

In the first stage of the study, an attempt was made to chemically modify the glass fibres in order to obtain the surface compound, acting as a source of benzene in the process of thermal decomposition. A diagram of the chemical modification of the fibre surface process is shown in Fig. 2.

The first step was to bind a suitable silane to fibres, in this case 3-chloropropyl–trimethoxysilane in order to introduce onto the surface of the fibres the connecting arm of suitable length and the –Cl functional group. The reaction was carried out in toluene at 80 °C. After finishing the reaction, fibres were washed sequentially with toluene, methanol and acetone solution and then dried in a vacuum evaporator. Then, the Schiff base (a quinoxaline derivative) was added to the prepared fibres. The reaction was conducted in dimethylformamide (DMF) at 80 °C for 12 h. After finishing the reaction, the fibres were again washed with solutions: DMF, methanol and acetone and then dried. The next step was the diazotization reaction of aniline by nitrate(III) sodium NaNO_2_ in the presence of hydrochloric acid. The reaction proceeded in an aqueous solution at a temperature of 0–5 °C. The formed diazonium salt was coupled with pre-modified fibres. The reaction proceeded at 0–5 °C, and the diazonium salt was added dropwise for 30 min to the fibres in a solution of pyridine. The prepared fibres were left for 2 h in a solution and then filtered off; washed successively with methanol, acetone and water; and dried in a vacuum evaporator.

In the next stage of the research, a procedure for modifying the surface of the carrier material to obtain a suitable surface compound acting as a source of toluene in the thermal decomposition process was developed.

The process of chemical modification proceeded in several stages, analogous to the process of chemical modification, to obtain a suitable surface compound acting as a source of benzene. The change occurred at the stage of diazotization. In the case of benzene, it was aniline, and the toluene phase was used for diazotization by using p-toluidine (p-methylaniline).

### Evaluation of optimal parameters for TD-GC-MS analysis

The information on the analytical procedure is summarized in Table 1. At the stage of the separation, the identification and quantification of analytes (benzene and toluene), using gas chromatography coupled with a mass spectrometer (GC-MS), was performed. In order to facilitate the installation of the glass fibres inside a steel tube for thermal desorption and to minimize the wall memory effect, cylindrical inserts made of poly(tetrafluoroethylene) (PTFE) were used. Each time, before glass fibre analysis and after each analysis, the value of the blank (the steel tube with installed inside an empty PTFE tube) of the TD-GC-MS system was checked in order to minimize the impact of the memory effect of the wall on the final result.

**Table 1:** Analytical procedure for the thermal decomposition of the surface compound chemically bound to the surface of glass fibres and quantitative determination of benzene and toluene using the TD-GC-MS system

Preparation of the glass fibre sample for the analysis	
• Placing a small amount of silanized glass wool (Alltech Associates Inc.) inside cylindrical inserts made of Teflon (Agilent Technologies) • Placing a predefined amount of glass fibres inside the Teflon tubes filled with silanized glass wool • Installation of Teflon tube with glass fibres inside the tubes made of polished stainless steel dedicated to thermal desorption	
The thermal decomposition of the surface compound which produces the desired analytes (benzene or toluene), a two-step thermal desorption technique	
Thermal desorber: Unity v.2, Markes International Ltd., Pontyclun, UK	The first stage of thermal desorption • Placing a steel tube with glass fibres in a thermodesorber oven • Heating the tube for 10 min at 280 °C (helium flow rate 50 ml/min) • Thermal decomposition of the surface compound, which produces the desired analytes, and their transport to micro-traps (glass tube filled with *Tenax TA* and *Carbotrap*, temp. 0 °C)
	The second stage of thermal desorption • Intensive heating of micro-trap to a temperature of 300 °C for 5 min • Transport of analytes from micro-trap to chromatography column (helium flow rate 1.5 ml/min) • Dispenser mode: splitless injection
Separation, identification and quantitative determination of analytes produced from thermal decomposition of the surface compound	
Gas chromatograph	Agilent Technologies 6890
Detector	Mass spectrometer (MS) (5873 Network Mass Selective Detector, Agilent Technologies)
The operating mode of the detector	Selected ion monitoring (SIM) Identifying ions (*m*/*z*) are 77 and 78 (for benzene) and 91 and 92 (for toluene)
The transmission line temperature	The transmission line temperature of GC-MS is 150 °C The ion source temperature is 230 °C, Quadrupole mass analyzer temperature is 150 °C
Capillary column	DB-5 ms (J&W), 30 m × 0.25 mm × 25 μm
Carrier gas	Helium (1.5 ml/min)
Temperature programme	50 °C for 5 min 7 °C min^−1^ to 150 °C 15 °C min^−1^ to 250 °C for 10 min

### Calibration of TD-GC-MS system

The calibration process of the TD-GC-MS system was performed using a reference gas mixture defined by the manufacturer (Linde Industrial Gases, Poland), a concentration of the analyte from the BTX group, which was 9.80 ± 0.2 ppm for benzene and 9.50 ± 0.2 ppm for toluene. For each analyte (benzene and toluene), two four-point calibration curves were created for two ranges of masses of analytes: from 3.1 to 15.6 ng and from 15.6 to 62.5 ng for benzene and from 3.6 to 17.9 ng and from 17.9 to 71.4 ng for toluene. The equations for the calibration curves were as follows:

- From 3.1 to 15.6 ng benzene: *y* = 2.90°10^5^ × + 6.75°10^5^; *R*^2^ = 0.999
- From 15.6 to 62.5 ng benzene: *y* = 2.46°10^5^ × + 1.57°10^6^; *R*^2^ = 0.995
- From 3.6 to 17.9 ng toluene: *y* = 3.06°10^5^ × + 6.59°10^5^; *R*^2^ = 0.999
- From 17.9 to 71.4 ng toluene: *y* = 2.58°10^5^ × + 1.85°10^6^; *R*^2^ = 0.993

where *y* is the MSD response expressed as a peak area (conventional units), *x* is the mass of analyte (ng), and *R*^2^ is the coefficient of determination.

## Results

### Homogeneity and stability studies

A homogeneity study was performed for randomly selected samples of chemically modified fibres coated with a surface compound, from which thermal decomposition was obtained: (a) benzene and (b) toluene.

One package of each material containing 20 optical fibres was modified with appropriate surface compound. Determination was made for 15 fibres (five fibres from three different packages), separately for benzene and toluene samples.

Stability studies were carried out for fibres stored at room temperature. The obtained results were compared with the results obtained for fibres stored under refrigeration (−18 °C) taken as a reference condition.

**Fig. 3:**
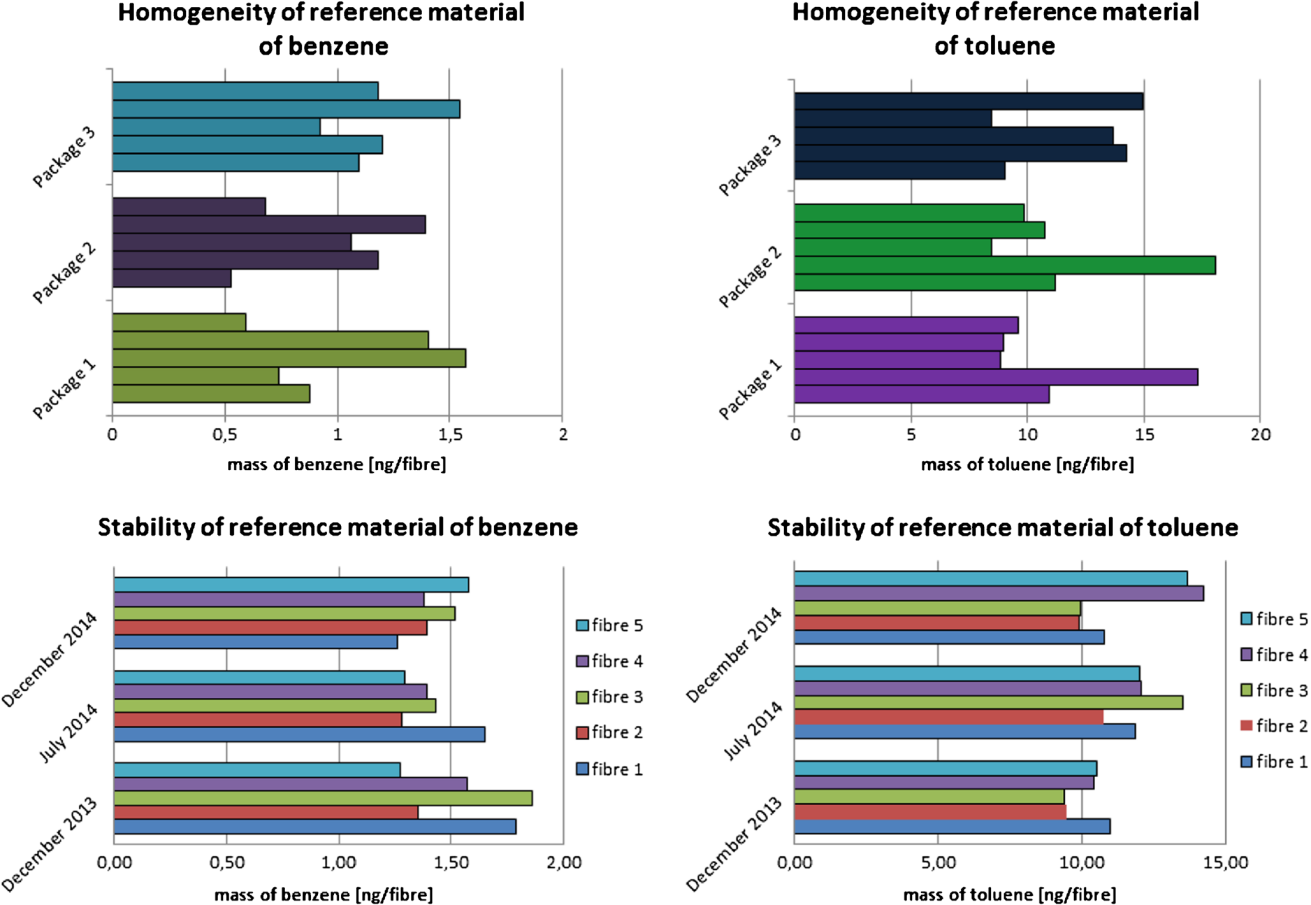
Determination of homogeneity and stability of matrix-free reference materials for benzene and toluene

The results obtained at the assessment stage of homogeneity and stability are shown in Fig. 3. Based on the obtained measurement results of benzene and toluene released from the surface of glass fibre segments, it is concluded that the materials prepared respectively for benzene and toluene are characterized by both within-unit and between-unit homogeneity.

In order to verify the accuracy of values obtained in the course of measurements, it is necessary to check the insignificance of the differences between the results. For this purpose, the method of calculating the ratio of the obtained results and the uncertainty of the determination was applied.

In order to verify the homogeneity of the material’s within-unit and between-unit homogeneity and to calculate the uncertainty in heterogeneity of materials, one-factor analysis of variance (one-way ANOVA) was conducted [19–21]. This statistical tool includes the criterion value (*F*_value_) and the critical value (*F*_critic_).

The information on the parameters characterizing ANOVA analysis applied to evaluate a set of obtained measurement results is summarized in Table 2.

**Table 2:** Statistical parameters of ANOVA analysis obtained at the stage of homogeneity study performed for candidates of reference materials of benzene and toluene

	Benzene		Toluene	
	Within-bottle homogeneity	Between-bottle homogeneity	Within-bottle homogeneity	Between-bottle homogeneity
Number of packages (*k*)	7	7	6	6
Number of samples (*i*)	5	5	5	5
Sum of squares (SS)	2.8247	0.8794	156.95	36.96
Number of degrees of freedom (*f*)	28 (*k* × *i* − *k*)	6 (*k* − 1)	24	5 (*k* − 1)
Variance of analysis (*V*)	0.1009	0.1466	6.54	7.40
Standard deviation (*S*)	0.32	0.38	2.56	2.72
Coefficient of variation (CV, %)	24	27	22.6	24
Participation in the uncertainty due to heterogeneity of the material (%)	43.3	56.7	46.9	53.1
*F*_value_ < *F*_critic_		1.31 < 2.44		1.13 < 2.62

On the basis of the obtained result, it is shown that the test criterion value (*F*_value_) is smaller than the critical value (*F*_critic_). The prepared *candidates* are characterized by good agreement among the units and packages. It was found that prepared candidates for reference materials for benzene and toluene are homogenous. It is proved by low values of such parameters as the coefficient of variation and standard deviation. It is also shown that the main element of uncertainty related to heterogeneity of prepared materials is connected with within-unit homogeneity of materials (83.5 % for benzene and 97.9 % for toluene).

For calculation of the uncertainty connected with heterogeneity of the material, Eq. (1) was used

1 $$ {S}_{\mathrm{bb}}=\sqrt{\frac{{\mathrm{MS}}_{\mathrm{among}}-{\mathrm{MS}}_{\mathrm{within}}}{n}} $$

where *S*_bb_ is the standard deviation between units, MS_among_ is the variance between packages, MS_within_ is the variance within package, and *n* is the number of replicates.

Moreover, for calculation of the combined standard uncertainty associated to homogeneity of materials, the repeatability of the method was taken into account (Eqs. (2) and (3))

2 $$ {S}_{\mathrm{r}}=\sqrt{{\mathrm{MS}}_{\mathrm{within}}} $$

3 $$ {u}_{\mathrm{h}}=\sqrt{S_{\mathrm{bb}}^2+{S}_{\mathrm{r}}^2} $$

where *u*_h_ is the combined standard uncertainty of homogeneity stage and *S*_r_ is the repeatability of measurement method.

The statistical analysis of the results obtained to assess the stability of the prepared reference materials was carried out using the method of calculating the ratio of the obtained results and the uncertainty of its determination.

The ratio of the average quantities of released benzene and toluene (*R*) was determined for the fibres stored over the 12 months. The parameter *R* was calculated for each of the periods in which the measurements were performed as a basis to assess the material’s stability.

The *R* value was calculated using the formula

4 $$ R=\frac{\overline{x_i}}{{\overline{x_i}}_{{}_{\mathrm{odn}}}} $$

The obtained results are presented in Table 3.

**Table 3:** Statistical evaluation of *R* parameter at the stage of stability study

	Period of time (months)	*R*	*U* (*k* = 2)
Toluene	6	0.84	0.19
	12	0.97	0.17
Benzene	6	1.11	0.18
	12	0.99	0.12

The calculated means for the compared series of results obtained for benzene and toluene do not differ in a statistically significant manner. This is due to the fact that in the range specified, value ± uncertainty of the calculated ratio of the appointment (*R* ± *U*) has a value of 1. That is why it can be concluded that the prepared material is stable in room temperature and its stability period is at least 12 months.

### Calculation of indicative value and uncertainty value

Indicative values for the prepared candidates for reference materials for benzene and toluene materials were calculated as the arithmetic mean of the measurement results obtained in the evaluation of the between-unit homogeneity of individual materials.

The uncertainty value of the indicative value was estimated as the expanded uncertainty of the indicative value combined to the uncertainties of homogeneity and stability tests.

6 $$ {U}_{\mathrm{indicat}}=\sqrt{u_{\mathrm{h}}^2+{u}_{\mathrm{stab}}^2} $$

7 $$ U={U}_{\mathrm{indicat}}\times k $$

where *U* is the expanded uncertainty and *k* is the coverage factor, for 95 % confidence interval (*k* = 2).

The contributions of uncertainties obtained at the stages of homogeneity and stability are presented in Table 4.

**Table 4:** Contributions of uncertainty obtained during homogeneity and stability study

Source of uncertainty	Benzene (ng/fibre)	Toluene (ng/fibre)
Uncertainty of homogeneity (*u*_h_)	0.45	3.7
Uncertainty of stability (*u*_stab_)	0.032	0.58

Benzene:

$$ \begin{array}{c}\hfill {M}_{\mathrm{benzene}}={X}_{\mathrm{avg}}\pm U\left(k=2\right)\hfill \\ {}\hfill x\pm U\left(k=2\right)=1.26\pm 0.91\kern0.5em \left(\mathrm{ng}/\mathrm{fibre}\right)\hfill \end{array} $$

Toluene:

$$ \begin{array}{c}\hfill {M}_{\mathrm{toluene}}={X}_{\mathrm{avg}}\pm U\left(k=2\right)\hfill \\ {}\hfill x\pm U\left(k=2\right)=11.3\pm 7.5\kern0.5em \left(\mathrm{ng}/\mathrm{fibre}\right)\hfill \end{array} $$

**Fig. 4:**
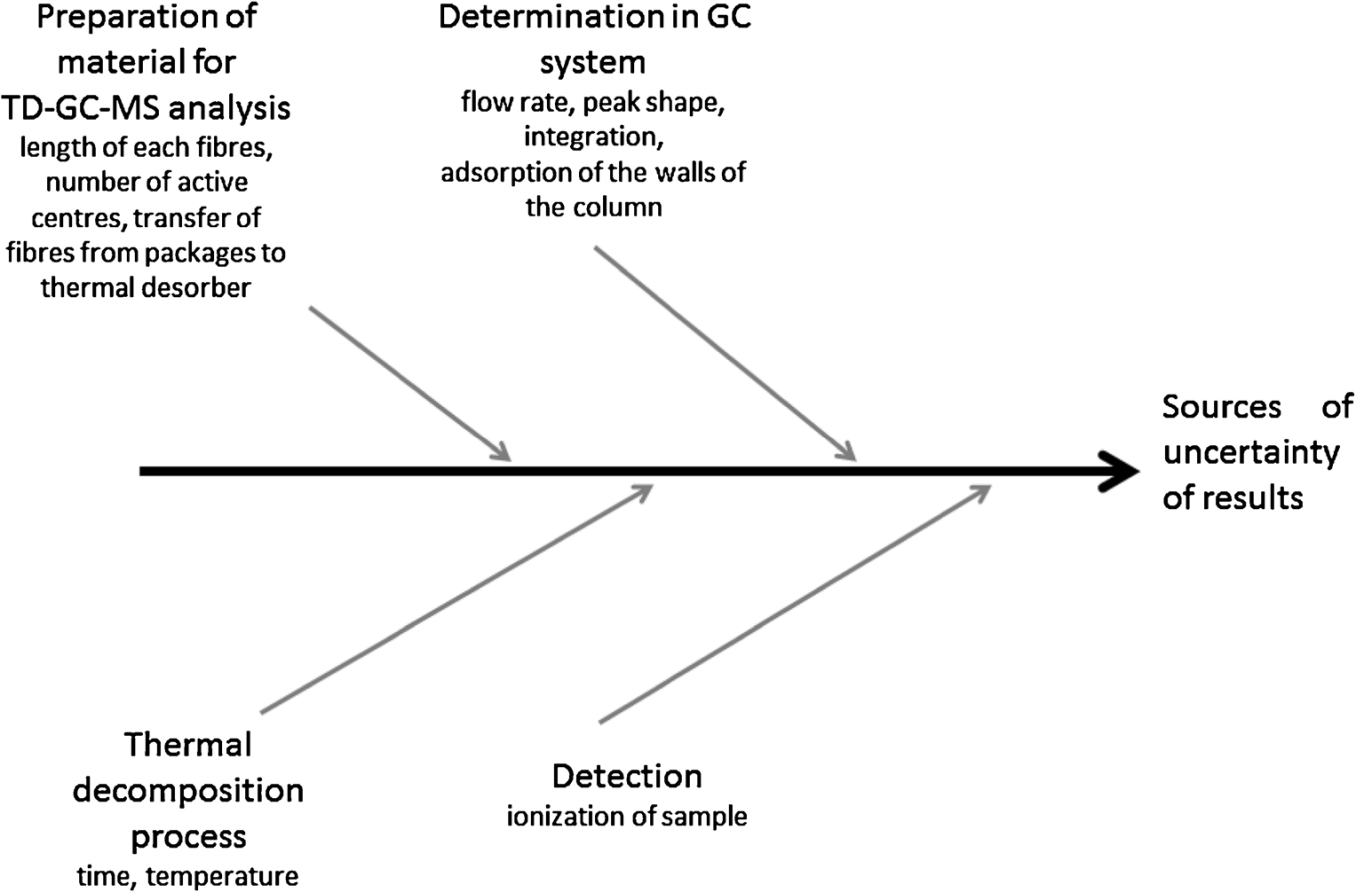
Source of uncertainty resulting from the applied analytical procedure

Prepared candidates meet all the requirements for reference materials. Accordingly, Fig. 4 contains the information on the possible errors’ sources associated with the use of candidates of matrix-free reference materials for benzene and toluene in everyday practice.

Analysis of uncertainty sources resulting from the applied analytical procedure can be stated that they do not affect the results obtained during the thermal decomposition of the prepared candidate reference materials.

## Conclusion

The obtained results confirmed the homogeneity and stability of the prepared batch of candidates for the title of matrix-free reference materials and resulted in the determination of the indicative value and estimation of the expanded uncertainty of the indicative value.

Prepared candidates for benzene and toluene reference materials are characterized by relatively high uncertainty values (72 % for benzene and 66 % for toluene). However, for microtrace and ultratrace concentration levels, the precision of the determination can be in the range of tens of percents due to the empirical equation of Horwitz [22].

As the result of this study, the possibility of applying glass fibres as a carrier for preparation of matrix-free benzene and toluene reference materials was confirmed.

Taking into account the commercially available types and forms of gaseous preparation reference materials, it should be noted that the proven solution is an alternative to the commonly used techniques for obtaining the reference gaseous mixtures in bottles.

The possibility of applying benzene and toluene reference materials in analytical studies in the form of glass fibres coated with a suitable carrier material allows the elimination of most of the disadvantages arising from the traditionally used static techniques for the preparation of reference materials, which include the following:

- The limited nature of the quantitative preparation of the mixture
- Time-consuming and inaccurate procedures
- Losses related to the processes of adsorption and condensation on the walls of the ventricular system for preparing gaseous calibration mixtures
- Failure to store an amount for reuse.

The simplicity of preparation and the much higher stability of the above-described candidate reference materials should be underlined as an advantage over bottled standard gaseous calibration mixtures.
